# Personal Perspectives on Plant Ribosomal RNA Genes Research: From Precursor-rRNA to Molecular Evolution

**DOI:** 10.3389/fpls.2021.797348

**Published:** 2021-12-21

**Authors:** Vera Hemleben, Donald Grierson, Nikolai Borisjuk, Roman A. Volkov, Ales Kovarik

**Affiliations:** ^1^Center of Plant Molecular Biology (ZMBP), University of Tübingen, Tübingen, Germany; ^2^Plant and Crop Sciences Division, School of Biosciences, University of Nottingham, Sutton Bonington Campus, Loughborough, United Kingdom; ^3^School of Life Sciences, Huaiyin Normal University, Huai'an, China; ^4^Department of Molecular Genetics and Biotechnology, Yuriy Fedkovych Chernivtsi National University, Chernivtsi, Ukraine; ^5^Laboratory of Molecular Epigenetics, Institute of Biophysics, Academy of Sciences of the Czech Republic, Brno, Czechia

**Keywords:** rDNA research history, rRNA precursor, rRNA processing, molecular evolution, epigenetics, polyploidy, hybridization, nucleolar dominance

## Abstract

The history of rDNA research started almost 90 years ago when the geneticist, Barbara McClintock observed that in interphase nuclei of maize the nucleolus was formed in association with a specific region normally located near the end of a chromosome, which she called the nucleolar organizer region (NOR). Cytologists in the twentieth century recognized the nucleolus as a common structure in all eukaryotic cells, using both light and electron microscopy and biochemical and genetic studies identified ribosomes as the subcellular sites of protein synthesis. In the mid- to late 1960s, the synthesis of nuclear-encoded rRNA was the only system in multicellular organisms where transcripts of known function could be isolated, and their synthesis and processing could be studied. Cytogenetic observations of NOR regions with altered structure in plant interspecific hybrids and detailed knowledge of structure and function of rDNA were prerequisites for studies of nucleolar dominance, epistatic interactions of rDNA loci, and epigenetic silencing. In this article, we focus on the early rDNA research in plants, performed mainly at the dawn of molecular biology in the 60 to 80-ties of the last century which presented a prequel to the modern genomic era. We discuss – from a personal view – the topics such as synthesis of rRNA precursor (35S pre-rRNA in plants), processing, and the organization of 35S and 5S rDNA. Cloning and sequencing led to the observation that the transcribed and processed regions of the rRNA genes vary enormously, even between populations and species, in comparison with the more conserved regions coding for the mature rRNAs. Epigenetic phenomena and the impact of hybridization and allopolyploidy on rDNA expression and homogenization are discussed. This historical view of scientific progress and achievements sets the scene for the other articles highlighting the immense progress in rDNA research published in this special issue of Frontiers in Plant Science on “Molecular organization, evolution, and function of ribosomal DNA.”

## Introduction

Cytologists in the twentieth century recognized the nucleolus as a common structure in all eukaryotic cells, using both light and electron microscopy. During the winter of 1931, Barbara McClintock observed that in interphase nuclei of maize the nucleolus was formed in association with a specific chromosomal region, normally located at the end of chromosome 6, which she called the nucleolar organizer region (NOR; McClintock, 1934). It took, however, almost 40 years before the composition of the NOR was deciphered (i.e., rRNA genes) with the aid of chromosome *in situ* hybridization techniques in the seventies (Gall, 1981). The molecular structure and function of the ribosomal RNA genes, which are located in or around the nucleolus, were analyzed after the structure of DNA, the triplet code and the central dogma “DNA makes RNA makes protein” were established. In early days of rDNA research, general molecular biological principles were being established rapidly. For example, experiments with cultured cells showed that radioactive amino acids were first polymerized in the cytosol, in association with ribosomes, which identified them as the subcellular sites of protein synthesis. The frog *Xenopus* became a crucial model system for studying the ribosomal RNA genes (rDNA) when Brown and Gurdon (1964) showed that the arrested development and eventual death of *Xenopus* anucleolate mutant embryos was due to their inability to make new rRNA. Birnstiel et al. (1966) demonstrated that these mutants lacked the rDNA, which was subsequently isolated from the wild type and shown to be composed of multiple copies of alternating 18S and 28S rDNA cistrons (Birnstiel et al., 1968). Distinct plant ribosomal DNA satellites had been noted around the same time by Matsuda and Siegel (1967) and characterized subsequently by Goldberg et al. (1972) and Bendich and Anderson (1974). In the mid to late 1960s the synthesis of nuclear-encoded rRNA was the only system in multicellular organisms where transcripts of known function could be isolated, and their synthesis and processing could be studied.

**Figure 1:**
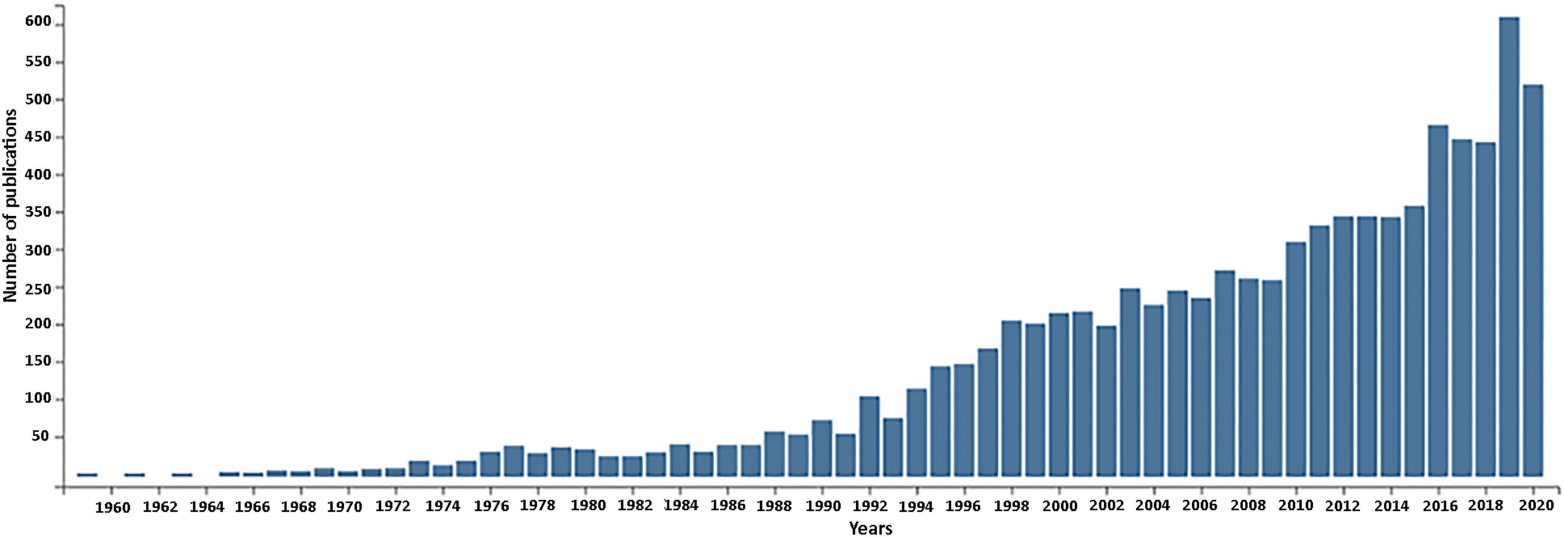
Publications related to plant nuclear rDNA research over the timespan of 1960–2020. The number of retrieved publications is shown in 2-year increments. The total number of publications returned by the Web of Science database was 8,948. Key words used for searched fields: rRNA *or* rDNA *and* plant with following filters: *no* animal, *no* fungal, *no* chloroplast (plastom), *no* mitochondrion.

In this retrospective article, we review research carried out in plants from the 60 to 80-ties of the last century focusing on nuclear encoded ribosomal RNA genes as a prequel to recent genomic activities (reviewed in Volkov et al., 2004, 2007; Layat et al., 2012; Sáez-Vásquez and Delseny, 2019; Appels et al., 2021 – this issue). The continuing rise in publications on rDNA in plants witnessed over the past 60 years (Figure 1) is testimony to its continuing importance.

## General Organization of Nuclear-Encoded 35S rDNA, rRNA Precursor, and 5S rDNA: The Beginning of Plant Molecular Research

**Figure 2:**
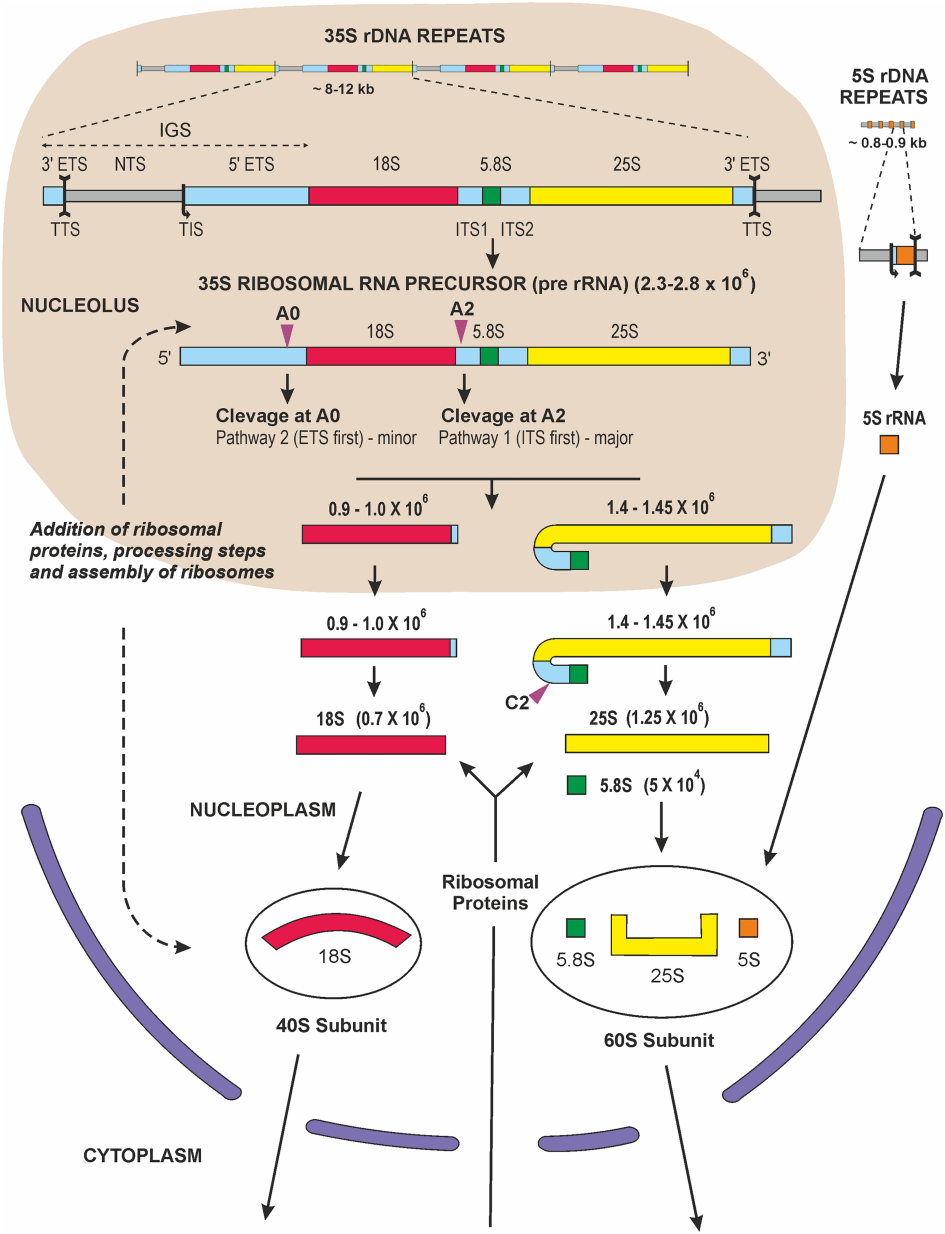
Graphic representation of ribosome biogenesis in eukaryotic cells (adapted from (Grierson, 1984; Sáez-Vásquez and Delseny, 2019). Transcription of rDNA requires RNA Pol I activity and a subset of general transcription factors. The primary transcript, precursor-rRNA (35S in plants and yeast or 45S in mammals), encodes three rRNAs and is first co-transcriptionally processed into the mature 18S, 5.8S, and 25S/28S rRNA. This processing steps involve multiple endonucleolytic and exonucleolytic cleavages (violet arrowheads) occurring in the nucleolus [A0 site or P site according to nomenclature of (Sáez-Vásquez and Delseny, 2019)], A2 site and the nucleoplasm (C2). The 18S rRNAs assemble with ribosomal proteins of the small 40S ribosomal subunit, RPSs, while 5.8S, 25S/28S and 5S rRNA assemble with ribosomal proteins, RPLs, forming the large 60S ribosomal subunit. The 5S rRNA is transcribed in the nucleoplasm by RNA Pol III and imported into the nucleolus. Assembly and transport of ribosomal particles from nucleolus to cytoplasm requires hundreds of specific 40S and 60S RBFs (Ribosome Biogenesis Factors). The 40S and 60S ribosomal subunits finally join to form translationally competent ribosomes in the cytoplasm. Sizes of individual rRNA molecules are in daltons.

Many scientists in the 1960s felt that the existence of the tough plant cell wall surrounding a small amount of cytoplasm and a large vacuole containing many secondary products made it very difficult to carry out molecular research on plants. This was certainly felt by some people in the Max Planck Society in Germany and also in the United Kingdom and United States, but plant molecular research began to flourish as methods to overcome these difficulties were developed. Research had been restarted rapidly at the University of Tübingen after the end of World War II. At the Botanical Institute of the University, most people were engaged in studies of the “Biological Clock” and circadian rhythms with Prof. Erwin Bünning (a leading plant physiologist and one of the founders of plant research in Tübingen, (Chandrashekaran, 2006), when a new assistant and later lecturer, Gerhard Richter, arrived. He had spent a research stay performing molecular research in the lab of the biochemist James F. Bonner in Pasadena, the United States. At that time in Tübingen, people from the Max Planck Institute were involved in codon studies and how messenger RNA transported the “Bauanleitung” (“contruction manual”) of proteins to the ribosomes, the sites for protein synthesis. So, the environment was prepared for molecular biology, and Gerhard Richter had no problem in convincing Prof. Bünning to establish new laboratories for this kind of research and, most importantly, for working with radioactive substances. Vera Hemleben (VH) thought it would be interesting to work with higher plants and to study nucleic acid synthesis in dark-grown seedlings of beans which could be cultivated under semi-sterile conditions. Other PhD students got involved, and ^32^P-phosphate radioactively labeled nucleic acids were isolated and separated on MAK [methylated albumin on Kieselgur (“silica”)] columns; we isolated the ribosomal RNA fractions and determined the GC-content of the 18S and 25S rRNA (Hemleben-Vielhaben, 1966). Other researchers, e.g., Joe Key now in Athens, Georgia, did similar work (Leaver and Key, 1970). VH decided later to work with *Lemna perpusilla*, the small aquatic monocot plant, which could be cultivated under completely sterile conditions. This was necessary for radioactive pulse-labeling and pulse-chase experiments to follow the fate of the newly synthesized RNA and to characterize the nuclear encoded rRNA precursor, which was 2.3 × 10^6^ Da in size (Hemleben, 1972). From the late 1960s the polycistronic transcription unit, the rRNA precursor, was studied in several different eukaryotic systems. Studies with animals established that the rRNA genes were transcribed as a polycistronic precursor, of variable size in different organisms (45S in humans and 35S in plants), which subsequently underwent endonucleolytic cleavage and methylation before being incorporated, together with ribosomal proteins, into nascent ribosome subunits and transported from the nucleolus to the cytosol (Figure 2). Cytosolic ribosomes, formed by 18S (in the small 40S ribosome subunit), 5.8S and 25S plus 5S rRNA (in the large 60S subunit) and ribosomal proteins, were isolated and were, of course, essential constituents in the popular wheat germ *in vitro* protein synthesis system.

In September 1968, Don Grierson joined Ulrich Loening’s laboratory in Edinburgh to study the synthesis of rRNA in primary leaves of mung bean seedlings (Grierson et al., 1970; Grierson and Loening, 1972, 1974). Ulrich had developed methods for extracting undegraded RNA, fractionating it on the basis of size in polyacrylamide gels and determining the molecular weights with great precision (Loening, 1969). Ulrich built electrophoresis tanks from Perspex sheets, with platinum wire electrodes, insisted on redistilling phenol for use in RNA extraction, recrystallizing acrylamide and using deoxygenated monomer solutions to get consistent polymerization and electrophoresis results. Disposable plastic ware and automatic microsyringes were the subject of dreams. Ulrich also devised a novel gel-scanner and apparatus for slicing gels and automated detection of radioactivity in the slices. These methods generated worldwide interest and attracted collaborators and many visitors to the laboratory. The synthesis and processing of a polycistronic precursor (pre-rRNA) was studied in several plants, including mung bean leaves and roots (Grierson et al., 1970; Grierson and Loening, 1974), pea roots and cultured artichoke cells (Fraser and Loening, 1974), carrot (Leaver and Key, 1970), cultured sycamore cells (Cox and Turnock, 1973). In general, the observations were similar: when seedlings, plant tissues, or organs were incubated with ^3^H-uridine or ^32^P-phosphate for short periods of time the radioactivity was incorporated into distinct macromolecular transcripts. On polyacrylamide gels these could be seen above a polydisperse array of RNA molecules, presumably mRNAs, nascent molecules and processing products. Molecular weight estimates for the largest molecules ranged from 2.3 to 2.8 × 10^6^ Da. At slightly later times, radioactivity was also detected in molecules of around 1.4 and 0.75 × 10^6^ Da and pulse-chase experiments, kinetics of accumulations and comparison of the nucleotide composition of these molecules all supported the conclusion that the initial transcript was a large polycistronic molecule that included one 18S, 5.8S and 25S transcript, together with “transcribed spacer” RNA. Subsequent ribonucleolytic cleavage gave rise to the immediate rRNA precursors, each slightly larger than the mature rRNAs. Aggregates and breakdown products of rRNAs could also be distinguished (Grierson and Loening, 1974), and electrophoresis under denaturing conditions in formamide gels gave lower molecular weight estimates of the size of the initial transcripts in carrot, parsley, and sycamore (Seitz and Seitz, 1979). The size characterization of individual pre-RNA molecules and the complexities of the rRNA maturation pathway stimulated further research. The processing of plant pre-rRNA, updated to show recent findings, is illustrated in Figure 2 and the enzymes catalyzing individual RNA cleavage steps (A0, A1, A2 and C2 sites) have now been identified (Tomecki et al., 2017). Moreover, plant small nucleolar RNA (snoRNAs) that are thought to take part in pre-rRNA cleavage events were identified (Brown and Shaw, 1998). It became clear that the cleavage events are compartmentalized, some occurring in the nucleolus (A0, and A2) with others take place in the nucleoplasm (C2; Figure 2). Finally, although key steps of plant pre-rRNA processing seem to be similar to that of other eukaryotes (Grierson, 1984) notable differences exist between yeast, plant and animal pre-RNA pathways. For example, the analysis of ribosome biogenesis in plants revealed two alternative processing pathways coexisting in plants (Weis et al., 2015; Sáez-Vásquez and Delseny, 2019). A major pathway 1 is initiated by ITS1 cleavage (A2 site, Figure 2) and subsequent removal of the 5'-ETS, which is comparable to the human processing pathway. Pathway 2 starts with the 5'-ETS removal (cleavage at the A0 site, Figure 2) followed by the ITS1 cleavage which leads to the separated assembly of the pre-40S and pre-60S ribosomal subunits. This pathway is reminiscent of rRNA processing in yeast. In addition, 5'-ETS processing is initiated by exoribonucleolytic trimming of the 5'-end by XRNs in *Arabidopsis thaliana* (Zakrzewska-Placzek et al., 2010).

Other rRNA transcription units were found transcribed from the DNA in chloroplasts, containing their smaller 16S and 23S rRNAs, components of the distinct 70S ribosomes, and a further class of ribosomes in the mitochondria, although their synthesis was not studied in such detail. Double labeling experiments showed a stable polycistronic precursor of rRNA in leaves, which was distinct from, and larger than that in roots (Grierson and Loening, 1972). This RNA species was subsequently shown to be synthesized by chloroplasts, although the conclusion that it represented a polycistronic chloroplast rRNA precursor was not unanimous (Hartley and Ellis, 1973; Grierson and Loening, 1974). This may have been because chloroplasts were believed to have been derived from blue-green bacteria during the course of evolution and prokaryotic rRNAs had been found to be monocistronic (Adesnik and Levinthal, 1969; Dahlberg and Peacock, 1971; Grierson and Smith, 1973; Seitz and Seitz, 1973). Of course, the similarity between the rRNAs of bacteria and chloroplasts had evolutionary significance, as is now widely recognized (Gray, 2017). Similarities between bacteria and these cellular organelles had been noted in the 19th and early 20th centuries but this idea was not given much credence until Lynn Margulis (then Lynn Sagan) published her account of what was described as “perhaps the first unified theory of eukaryogenesis,” proposing, as is now widely accepted, that “mitochondria and plastids might have originated endosymbiotically from prokaryotic progenitors” (Gray, 2017).

The discovery and application of restriction enzymes by Werner Arber, Daniel Nathans, and Hamilton O. Smith, who were jointly awarded the Nobel Prize in Physiology or Medicine in 1978 and the work of Sir Kenneth Murray (department of Molecular Biology, University of Edinburgh), in combination with development of other new technologies for transformation of bacteria with foreign DNA, paved the way for gene cloning and experimental gene transfer between organisms. In Tübingen, now at the Genetics department, the VH group planned to study gene transfer in higher plants with the genetically well-defined *Matthiola incana*. Therefore, highly ^3^H-labeled total DNA was supplied to plant seedlings, and we found integration of this foreign DNA into the nuclei (Hemleben et al., 1975; Leber and Hemleben, 1979). We reported these results at the first Plant Molecular Biology Conference in Liege in 1974. Here, we met Don Grierson, and we organized a 1-year project in 1975/76, supported by an EMBO Long-Term Fellowship to DG. During a meeting in Edinburgh (a summer school run by Ulrich Loening and the late Max Birnstiel in Edinburgh in 1975), we had learned how to separate DNA on Actinomycin D-CsCl gradients and to separate the often highly repeated rRNA genes (rDNA) from the main-band DNA in animal systems (see Birnstiel et al., 1968). Don had also done this with mung beans for his PhD (Grierson, 1972), and this opened up the possibility to purify plant rDNA (Grierson and Hemleben, 1977; Hemleben et al., 1977) and later to characterize it by restriction enzyme analysis, which we carried out firstly during a research stay at Joe Key’s lab in Athens/Georgia in 1978 (Friedrich et al., 1979).

The DNA-content of animals and plants (for plants see: Nagl et al., 1979; Wenzel and Hemleben, 1982a; Michael and Van Buren, 2020) appeared to vary enormously, and the question was: What is the explanation for repetitive genome components and which sequences are redundant? The mature, purified and radioactively labeled rRNAs were used as probes in classical liquid phase hybridization experiments with genomic DNA, showing often enormously high numbers of the tandemly repeated genes for the 18S, 5.8S and 25S (Matsuda and Siegel, 1967; Bendich and Anderson, 1974; Ingle et al., 1975; Wenzel and Hemleben, 1982a). Matsuda and Siegel (1967) further showed that the amount of rDNA cistrons (units) in the nuclear DNA varied among tobacco, pumpkin, pinto beans and Chinese cabbage plants over a 10-fold range. Therefore, it became clear that, besides the highly repetitive satellite DNA, the tandemly arranged and highly redundant rRNA genes contribute to this variability (Marazia et al., 1980; Rogers and Bendich, 1987). Our first chromatin and methylation studies with *Matthiola incana* and *Brassica pekinensis* showed that most of the rRNA genes were not transcriptionally active. The rDNA-containing chromatin was not accessible to DNase I digestion, and most of the rRNA genes appeared highly methylated (Leber and Hemleben, 1979; Leweke and Hemleben, 1982; Wenzel and Hemleben, 1982b). At that time, this gene silencing phenomenon was not yet widely called “epigenetics,” although there was a department of Epigenetics established at the Edinburgh University. Transcriptional regulation of nuclear encoded rRNA genes by methylation and demethylation, respectively, was observed also by other researchers (Flavell et al., 1988; Thompson and Flavell, 1988). In Tübingen, we were working with *Cucurbitaceae* species, which were known for an enormously high number of ribosomal RNA genes (Bendich and Anderson, 1974) and in the 1990s a PhD student Ramon Torres-Ruiz was able to identify the pattern and degree of methylation in the rDNA of *Cucurbita pepo* (Torres-Ruiz and Hemleben, 1994).

The nuclear ribosomal DNA could, as described, be separated from the main nuclear DNA by Act D-CsCl gradient ultracentrifugation followed by restriction enzyme mapping. This enabled in the early 1980s cloning of plant rDNA using specific gene probes for the 18S, 5.8S, and 25S rRNA and for the internal transcribed (ITS1 and IT2), external transcribed (ETS) and the non-transcribed (NTS) regions of the intergenic spacer (IGS; Figure 2), which allowed subsequent rDNA sequencing. Early on, large DNA fragments, especially those containing internal repeated DNA elements (subrepeats) identified later in the 35S rDNA IGS (35S IGS), were difficult to clone in plasmid vectors, but they could be analyzed by restriction endonucleases and often showed length heterogeneity even in single individuals (Ganal and Hemleben, 1986; Rogers et al., 1986; Yokota et al., 1989). Later, we and others were able to clone the complete large IGS and to characterize this region by DNA sequencing using at that time the radioactive sequencing methods (Sanger et al., 1977). Of course, the polymerase chain reaction (PCR) developed by the biochemist Karl Mullis, who in 1993 shared the Nobel Prize in Chemistry, facilitated enormously this procedure. Length heterogeneity of the rDNA repeats was due mostly to different numbers of subrepeats in the 35S IGS upstream or downstream of the transcription initiation site, TIS (Appels et al., 1986; Ganal et al., 1988; Rathgeber and Capesius, 1990; Borisjuk and Hemleben, 1993; King et al., 1993; Zentgraf and Hemleben, 1993; Figure 2, upper part). Interestingly, it appeared that in some plants (e.g., some *Vigna* sp.) these repeated elements of the 35S IGS formed independent highly amplified satellite DNA genome components (Unfried et al., 1991; Macas et al., 2003; Lim et al., 2004b; Jo et al., 2009; Kirov et al., 2018). In contrast, 35S DNA amplification of satellites within the 5S rDNA loci is rare. Nevertheless, a 170-bp satellite sequence (termed jumper) apparently invaded 5S rDNA in the evolutionary history of the *Phaseolus* genus (Ribeiro et al., 2017). Of note, only a single satellite monomer is found in the 5S IGS while there may be thousands of copies outside of 5S rDNA loci, forming pericentromeric and subtelomeric domains. This may suggest that the rDNA intergenic spacers have relatively frequently hosted non-coding satellites but their expansion is limited to one or a few copies, probably due to selection constrains imposed on spacer lengths.

In chromosomes, the 5S rRNA genes occur either as long tandem repeats of regularly spaced units (S-type arrangement) or as solitary insertions within the 35S rDNA intergenic spacer (L-type arrangement). The S-type arrangement is the most frequent organization of 5S rDNA in angiosperms (Ellis et al., 1988; Hemleben and Werts, 1988; Scoles et al., 1988; Röser et al., 2001) while, so far, the L-type arrangement has been detected only in some member of the Asteraceae family (Garcia et al., 2009, 2010; Souza et al., 2019). The L-type arrangement is more typical for plants that diverged early during angiosperm evolution (Capesius, 1997; Wicke et al., 2011) and some groups of gymnosperms (Galian et al., 2012; Garcia and Kovarik, 2013). The 5S rDNA was apparently invaded by an LTR transposon in the early evolutionary history of angiosperms, giving rise to Cassandra retrotransposons (Kalendar et al., 2008), which are now widespread in modern species. Truncated incomplete copies of 35S rDNA seem to be scattered in genomes of both plants (Tulpová et al., 2020) and animals (Robicheau et al., 2017) and likely represent remnants of former NORs.

## Transcriptional Regulation of rRNA Genes

The nuclear encoded 18S, 5.8S, and 25S ribosomal RNA genes were already known in yeast and animals to be transcribed by RNA polymerase I, and this was confirmed in plants in Joe Key’s lab (Lin et al., 1976) and in Tübingen (Grossmann et al., 1979, 1980). Functional studies identified the putative transcription initiation (TIS) and transcription termination sites (TTS) for plant rDNA (Delcasso-Tremousaygue et al., 1988; Gerstner et al., 1988; Gruendler et al., 1989; Schiebel and Hemleben, 1989; Zentgraf and Hemleben, 1992, 1993; Figure 2, upper part). In addition, the repeated elements upstream or downstream of the TIS obviously had an enhancer function (reviewed in Hemleben and Zentgraf, 1994). Under the current view, the nucleoprotein complex responsible for transcription initiation of 45S (35S) rRNA is composed of numerous protein components (Nucleolar Remodelling Complex (NoRC), UBF, histone acetyltransferases, helicases, and RNA polymerase I, among others) and several species of noncoding RNA (Bersaglieri and Santoro, 2019; Yan et al., 2019). The RNA polymerase I holoenzyme has been purified to apparent homogeneity by biochemical approaches, maintaining the capacity to initiate rDNA transcription (Sáez-Vásquez and Pikaard, 2000). Nucleolin, a relatively abundant nucleolar structural protein, seems to be involved in selection of rDNA variants for transcription in Arabidopsis (Pontvianne et al., 2010).

The 5S rRNA genes are transcribed by RNA polymerase III and their promoter elements and termination sites were putatively described for plants (Hemleben and Werts, 1988). Transcriptional regulation of the multigenic 5S rDNA in *Arabidopsis* has been extensively studied in Sylvette Tourmentes’s laboratory (University of Clermont-Ferrand, France), confirming the originally described 5S rRNA internal and external elements involved in regulation of the gene’s transcription (reviewed by Layat et al. (2012). Later, in Roman Volkov’s laboratory, conservation of the putative external promoter elements in the 5S rDNA intergenic spacer (5S IGS) was demonstrated for several families of angiosperms (Volkov et al., 2001, 2017; Tynkevich and Volkov, 2014, 2019; Ishchenko et al., 2018, 2020).

While the major advances in our knowledge of rDNA regulation were achieved in yeast (Moss, 2004) and animals (rat, mouse and *Xenopus*; (Grummt et al., 1985; Pikaard and Reeder, 1988), the research on plant rDNA also made significant progress over the years (Weis et al., 2015; Sáez-Vásquez and Delseny, 2019). Now it is widely accepted that in addition to transcriptional regulation of individual rDNA repeat units, the entire rDNA arrays (NOR) are targets of regulation as exemplified by studies in *Arabidopsis* (Mohannath et al., 2016) and wheat (Handa et al., 2018). The findings described recently by (Sims et al., 2021), sequencing entire rDNA arrays and deciphering their higher structure promise further deeper insights into the functional organization and molecular regulation of plants rDNA loci. By applying a combination of long- and short-read sequencing the authors revealed clustering of rDNA domains in *Arabidopsis* NOR2 and expression of several variants of rRNAs with their tissue-specific integration into active ribosomes (Sims et al., 2021). In leaf tissue of the ecotype Columbia-0 (reference genome), the rDNA of NOR4 on chromosome 4 is usually more active than that on chromosome 2 (NOR2; Chandrasekhara et al., 2016). However, NOR4 is not always dominant and many natural populations of *Arabidopsis thaliana* show considerable epigenetic variability, i.e., dominant expression of NOR4, NOR2, or codominant expression of both loci (Rabanal et al., 2017).

The duckweed species *Spirodela polyrhiza* and *S. intermedia* might emerge as a promising new model to study rDNA regulation (in addition to *Arabidopsis*) because of their compact rDNA loci, composed of no more than a hundred 35S repeated units (Michael et al., 2017; Hoang et al., 2020), even fewer than the 100–200 rDNA copies in yeast (Salim et al., 2017), and an order of magnitude lower than rRNA gene copies in other plants (Wang et al., 2019). The copy number of 5S rDNA is estimated to be about 170 in *Landoltia punctuata* (Chen et al., 2021) – this issue. This exceptionally low copy number makes the duckweed rDNA locus relatively simple to access applying the third-generation sequencing platforms for ultra-long sequencing reads (Jung et al., 2019).

## Molecular Evolution of rDNA Loci

Although it was initially believed that the rRNA genes are quite conserved, it turned out in the 1980s that the regions of the 5' and 3’ ETS, NTS, and ITS1 and 2 are characterized by a huge intra- and interspecies variability. This made them highly suitable as markers for our phylogenetic and molecular evolution studies at population or interspecies levels (Torres et al., 1989; Grebenstein et al., 1998; Jobst et al., 1998; Volkov et al., 2003, 2010; Denk et al., 2005; Grimm et al., 2005; Komarova et al., 2008; Schlee et al., 2011). Table 1 summarizes individual 35S rDNA subregions used for phylogenetic markers.

**Table 1:** Characteristics of individual subregions of plant rDNA units and their relevance for phylogenetic analysis.

Feature	Coding regions (5S, 5.8S, 18S, 25S rRNA)	35S-NTS^a^	5’ ETS^b^	ITS1/ITS2	5S-NTS^c^
Tempo of evolution	Slow	Extremely fast	Fast	Fast to moderate	Fast to moderate
Subrepeated structure	No	Frequent	Occasionally	Exceptional	Rare
Resolution power in phylogenetic studies	Order/family	Species/subspecies/cultivars/populations	Genus/species	Genus/species	Genus/species
Utility for interspecific hybrids identification	No	Intermediate	Good	Good	Excellent

a *A part of the 35S rDNA intergenic spacer located between the transcription termination site (TTS) and the transcription initiation site (TIS - see* Figure 2*)*.

b *A part of the 35S rDNA intergenic spacer located between the TIS and the 18S rRNA gene*.

c *Non-transcribed intergenic spacer of variable size between tandemly arranged 5S rRNA genes*.

Similarly, the 5S IGS appeared very useful for clarifying phylogenetic relationships at low taxonomic levels (Röser et al., 2001; Volkov et al., 2001; Denk and Grimm, 2010; Tynkevich and Volkov, 2019; Ishchenko et al., 2021) and for identification of interspecific hybrids (Garcia et al., 2020). This contrasts with the really strongly conserved sequences of the mature 5S, 5.8 S, 18S, and 25S rRNA coding regions. However, variable segments of 18S and 25S coding regions (“expansion segments”) evolve faster than the conserved stems. These features of rRNA coding regions can be used for phylogenetic studies particularly at higher taxonomic levels (Poczai and Hyvonen, 2010; Soltis and Soltis, 2016). The secondary structure of rRNA transcripts is another layer of phylogenetic information in addition to primary sequence (Noller et al., 1981; Selig et al., 2008). Currently, a public database of ITS2 secondary structure models comprise more than 80 thousand sequences (Selig et al., 2008). Consequently, various rDNA regions have been studied worldwide in the 1990s until the present day as phylogenetic markers in population, species, genus, and higher systematic order studies and helped to solve the phylogenetic relationships between organisms. Public databases storing biological information about rDNA loci (Szymanski et al., 1998; Selig et al., 2008; Cantara et al., 2011; Garcia et al., 2012; Quast et al., 2012) represent a valuable source for structural, functional and phylogenetic studies.

In the 1990s Nikolai (NB) and Ljudmilla Borisjuk and later Roman Volkov (RV) and Irina Panchuk from the Ukraine joined the VH laboratory, NB and RV as Alexander v. Humboldt fellows. They had already started to study plant rDNA, and we had a very successful cooperation over the years working mostly on several genera of Solanaceae (Borisjuk et al., 1994, 1997; Volkov et al., 1999a,b; Komarova et al., 2004). In 1983–1985, RV and NB worked in the group of Andrey S. Antonov at Moscow University (Russia). NB worked on the characterization of genomes of Solanaceae and Brassicaceae somatic hybrids generated by protoplast fusion in the Lab of Yuri Gleba in Kyiv (Ukraine). RV was interested in describing rearrangements of repeated sequences in natural allopolyploids, particularly in the genus *Nicotiana*, which includes several allopolyploids and aneuploids. These young researchers decided to perform the 35S rDNA restriction mapping for several artificial and natural allopolyploids. They used as probes for Southern hybridization 18S and 25S rRNA from maize and a fragment of 25S coding sequence of lemon isolated by Volodymyr Kolosha (Pushchino, Russia) under the supervision of Tengiz Beridze (Georgia). Mapping experiments revealed that generally the interspecies and inter-tribal hybrids obtained by protoplast fusion or sexual crossing inherited a combination of parental rDNA (Gleba et al., 1988; Borisjuk and Miroshnichenko, 1989; Miroshnichenko et al., 1989). Additionally, a novel class of rDNA repeats was found in somatic hybrids between distantly related *Nicotiana* and *Atropa* (Borisjuk et al., 1988). Later on, this observation let to the discovery of an “amplification promoting sequence” (APS) within the tobacco 35S IGS. The cloned APS element apparently increased the copy number of linked reporter genes in transgenic experiments resembling the origin of DNA replication (Borisjuk et al., 2000). It transpired that the structure of the *Nicotiana* 35S IGS is highly complex, bearing repetitive subregions which apparently account for species-specific differences in rDNA structure (Volkov et al., 1996; Borisjuk et al., 1997). Genomic analysis showed numerous SNPs (single nucleotide polymorphisms) in the tobacco 35S IGS, which indicated that the mutation rate in that region may be faster than that of coding regions, arguing for variable selection pressures acting on different parts of the rDNA unit (Lunerova et al., 2017).

## The Fate of rDNA in Solanaceae Hybrids and Allopolypoids

The multigene families in both plants and animals reveal high levels of intra-species homogeneity and inter-species diversity. These features underlie the concept of concerted evolution put forward by geneticists in the second half of the last century (Brown et al., 1972; Zimmer et al., 1980; Dover, 1982). It has become clear that concerted evolution (i.e., homogenization) processes affect nearly all repeated families including non-coding satellites and rDNA. The tandemly arranged rDNA represent a textbook example of concerted evolution since their hundreds of units show little or no intragenomic variation (reviewed by (Eickbush and Eickbush, 2007; Nieto Feliner and Rossello, 2012). The presence of rDNA arrays that are homogeneous for different variants in interbreeding populations of *Drosophila melanogaster* indicated that there is little recombination between the arrays while there might be intensive recombination within the arrays, leading to their overall homogeneity (Schlotterer and Tautz, 1994).

Towards the end of the last century the VH group explored the Solanaceae family (nightshades) which includes many economically important crops such as tomato, potato and tobacco. Within the family, the *Nicotiana* genus, whose center of diversity is Latin America, contains at least 50 allopolyploids of different ages and genome compositions. *Nicotiana tabacum* (tobacco) is, perhaps, the most well-known allotetraploid (2n = 4x = 48, genome composition SSTT) and has long been a favorite model for plant genetic studies (Goodspeed, 1954) including transgenosis and chromosome evolution (Manoharlal et al., 2019; Dodsworth et al., 2020). It is a relatively recent (*ca.* 0.1 myrs old; Lim et al., 2007) allopolyploid originating from hybridization of progenitor species close to *Nicotiana sylvestris* (2n = 2x = 24, S genome) and *Nicotiana tomentosiformis* (2n = 2x = 24, T genome). Its parental S- and T-genomes are relatively intact with few intergenomic translocations (Lim et al., 2004a). The question was: What is the fate of parental 35S rDNAs in tobacco? Are they intact or have they been modified by allopolyploidy?

In order to clarify the fate of parental 35S rDNA in the genome of *N. tabacum*, NB and RV decided to sequence the 35S IGS regions. From 1990 to 1992, with the support of the Alexander v. Humboldt Foundation, NB worked in the group of VH, who by then was a well-known leader in rDNA research. Here he performed restriction mapping of numerous *Solanum* species and other Solanaceae (Borisjuk et al., 1994) and cloned and sequenced the 35S IGS of *S. tuberosum* (Borisjuk and Hemleben, 1993) and *N. tabacum* (Borisjuk et al., 1997). During this time VH’s lab used RFLP (restriction fragment length polymorphism) for characterizing artificial somatic hybrids of *Solanum tuberosum* and various wild *Solanum* species produced by protoplast fusion in the lab of Prof. Helga Ninnemann by Dr. Lieselotte Schilde-Rentschler, mainly to introduce pathogen resistant characters into the cultivated potato (Schweizer et al., 1993). Therefore, the *Solanum* research of NB and RV was very complementary to the hybrid identification research. Interestingly, the rDNA of one fusion partner disappeared very quickly (see below for *Nicotiana*).

RV obtained an Austrian exchange service Research Fellowship, and in 1993 he went to the lab of Prof. Dieter Schweizer (Department of Cytology and Genetics, University of Vienna), where the 35S IGS of *Arabidopsis thaliana* had recently been sequenced and characterized (Gruendler et al., 1989). In Dieter Schweizer’s laboratory, RV cloned and sequenced 35S IGS of *N. sylvestris* and *N. tomentosiformis* (Volkov et al., 1996, 1999b).

In 1996, RV received the AvH Research Fellowship, moved to Tübingen and joined the VH group in order to investigate further the rDNA in Solanaceae. Comparative analysis of the 35S IGS sequences of N*. tabacum*, *N. tomentosiformis* and *N. sylvestris* allowed the molecular evolution of parental rDNA in the genome of *N. tabacum* to be deduced. Strikingly, only units similar to the paternal *N. tomentosiformis* genome (T-genome) were cloned from the tobacco genome (Volkov et al., 1999b), while clones from the maternal parent were not recovered, indicating elimination of the S-genome rDNA. It was found that the rDNA repeats of *N. tabacum* originated from *N. tomentosiformis*, which involved reconstruction of subrepeated regions in the 35S IGS upstream and downstream of the transcription initiation site. These cloning results well-resonated with the non-additivity of tobacco 35S rDNA restriction fragments observed in previous Southern hybridization experiments (Borisjuk et al., 1989; Miroshnichenko et al., 1989; Volkov et al., 1991; Kovarik et al., 1996). Clearly, thousands of parental rDNA units were overwritten by novel hybrid-specific units in relatively short evolutionary time (<100 thousand years; Borisjuk et al., 1997; Volkov et al., 1999a,b). Molecular cytogenetics approaches carried out by Andrew Leitch’s group (University of London) revealed that the number of tobacco rDNA loci is additive, i.e., there is a single locus in the T-genome and three loci in the S-genome (Lim et al., 2000). Only a small number of unconverted and highly methylated S-genome units were detected in the tobacco genome by molecular methods. In 2017 Jana Lunerova (AK group) using locus-specific FISH probes addressed the question of chromosomal localization of these transcriptionally inactive rRNA genes (Lunerova et al., 2017). It appeared that the residual (about 8% of total rDNA) S-genome units are located on a small acrocentric chromosome S12 while active homogenized genes are located on chromosomes T3, S10, and S11. Of note, the S-genome rDNA loci were found in the variety SR-1 and wild tobacco collected by Sandy Knapp (Natural History Museum London) in Bolivia, but not in the variety 09555 (Kovarik et al., 2004). Thus, the process of cultivation and high inbreeding may potentially influence the behavior of rDNA in allopolyploids. The Leitch’s and AK’s groups further confirmed partial and complete homogenization of parental rDNAs in another two *Nicotiana* allotetraploids, *Nicotiana rustica* (Indian tobacco, 2n = 4x = 48; Matyasek et al., 2003) and *Nicotiana arentsii* (2n = 4x = 48), respectively (Kovarik et al., 2004). Hence genetic interactions of rDNA loci seem to be a rather general feature of rDNA evolution in *Nicotiana* allopolyploids.

Fulnecek et al. (2002) investigated the structure of 5S rDNA loci which occurs separately from 35S rDNA loci in most plant genomes (Hemleben and Grierson, 1978; Garcia et al., 2016). Locus-specific FISH together with pulsed-field gel electrophoresis mapping showed that parental 5S rDNA arrays remained relatively intact and were inherited at expected ratios in tobacco allotetraploid. Therefore, in contrast to 35S rDNA, the 5S rDNA loci do not genetically interact in tobacco allotetraploids. The reason for higher genetic stability of 5S rDNA compared to 35S rDNA is not fully understood. However, 5S rDNA is highly methylated (more than the genome average; Fulnecek et al., 1998), while 35S rDNA units contain many undermethylated sites particularly in intergenic spacers (Torres-Ruiz and Hemleben, 1994). Hypomethylated sites in 35S IGS were also observed in other species including *Arabidopsis* (Earley et al., 2006), cucumber (Torres-Ruiz and Hemleben, 1994), potato (Komarova et al., 2004) and wheat (Sardana et al., 1993). It is possible that apart from transcription regulation these undermethylated sites might be important for adopting chromatin conformation favorable to recombination processes (Kovarik et al., 2008).

Volkov et al. (2017) applied a combination of karyological and molecular methods to investigate chromosomal localization, molecular organization and evolution of 5S and 35S rDNA in *Atropa belladonna* (Solanaceae), one of the oldest known flowering plant allohexaploids. Intensive sequence homogenization between three pairs of 35S rDNA loci on separate chromosomes was found, presumably inherited from tetraploid and diploid ancestor species. Only four out of six 35S rDNA sites appeared transcriptionally active, demonstrating nucleolar dominance. For 5S rDNA, three size variants of repeats were detected, with the major class represented by repeats containing all functional 5S IGS elements required for transcription, whereas intermediate and short length repeats contained defects both in the spacer and coding sequences. The functional 5S rDNA variants are nearly identical at the sequence level, pointing to their origin from a single parental species. Localization of the 5S rRNA genes on two chromosome pairs further supports uniparental inheritance from the tetraploid progenitor. The data demonstrate complex evolutionary dynamics of rDNA loci in allohexaploid species of *Atropa belladonna*. The high level of sequence unification revealed in 5S and 35S rDNA loci of this ancient hybrid species have been seemingly achieved by different molecular mechanisms.

## Nucleolar Dominance in Solanaceae Hybrids and Allopolyploids

Nucleolar dominance is an epigenetic phenomenon in which one parental array is inactivated in interspecific hybrids and allopolyploids. It was first described at the cytological level by Navashin (Navashin, 1934), who observed that in interspecific hybrids of *Crepis* (Asteraceae) only chromosomes of one crossing partner carried secondary constrictions at metaphase. This chromosomal region was not lost in hybrids but could be reactivated to produce normal nucleoli in hybrids with a different crossing partner. Experiments with epigenetic inhibitors performed in plants towards the end of the last century established that histone deacetylation and DNA methylation pathways interact in a self-reinforcing mechanism, maintaining silencing of partner rDNA units in hybrids (Chen and Pikaard, 1997a; Chen et al., 1988). The VH and AK groups investigated nucleolar dominance from different perspectives, asking questions about the influence of structural features of the 35S IGS, cytosine methylation of rDNA units and developmental stability of nucleolar dominance. To address these questions, they used well-defined natural and synthetic *Nicotiana* and *Solanum* (Solanaceae) allotetraploids. A comparison of 35S rDNA organization in several *Solanum* species revealed (Borisjuk and Hemleben, 1993; Borisjuk et al., 1994) that *S. lycopersicum* (tomato), *S. tuberosum* (potato) and wild species *S. bulbocastanum* possess 35S IGS of nearly identical length but contain different number of subrepeats up- and downstream of the TIS. Accordingly, VH and RV suggested using these species to elucidate the presumptive role of subrepeated elements in nucleolar dominance. In 1998, Nataliya Komarova from RV’s group moved from Ukraine to VH’s lab, where she studied expression of parental 35S rDNA in *Solanum lycopersicum* x *S. tuberosum* and *S. tuberosum* x *S. bulbocastanum* artificial somatic alloploids produced by protoplast fusion and back-crossed lines, which were kindly provided by E. Jacobsen and H.J. de Jong (Wageningen University, The Netherlands) and by L. Schilde-Rentschler and H. Ninnemann (University of Tübingen, Germany). It appeared that an expression hierarchy exists: In leaves, roots, and petals of the respective allopolyploids, rDNA of *S. lycopersicum* dominates over rDNA of *S. tuberosum*, whereas rDNA of *S. tuberosum* dominates over that of the wild species *S. bulbocastanum*. Also, in a monosomic addition line carrying only one NOR-bearing chromosome of tomato in a potato background, the dominance effect was maintained. These results demonstrated that there is possible correlation between transcriptional dominance and number of conserved elements downstream of the transcription start in the *Solanum* rDNA (Komarova et al., 2004). The authors proposed that this sequence motif could be a recognition site for DNA-interacting proteins involved in modulation of rDNA transcription (Borisjuk et al., 1997; Volkov et al., 2003). Remarkably, no correlation between the number of upstream subrepeats and differential transcription/silencing of 35S rDNA in *Solanum* allopolyploids was found. The latter contrasts with observations made in the allohexaploid wheat (AABBDD), where longer B-genome units containing more upstream subrepeats are active while the shorter units located in the D genome are usually inactive (Sardana et al., 1993). Units bearing longer upstream elements also seem to be dominant in recently (<100 years) formed *Tragopogon allotetraploids* (Matyasek et al., 2016). In contrast, sexual F1 hybrids resulting from crossing of *N. sylvestris* x *N. tomentosiformis* plants and a synthetic tobacco line show codominance (Dadejova et al., 2007), despite apparent differences in the 35S IGS structure of progenitor units. Thus, it seems that the role of 35S IGS repeat elements in regulation of rDNA expression varies from system to system. Certainly, a direct proof for an enhancer/silencing role of these elements, as shown in *Xenopus laevis* 35S IGS (Caudy and Pikaard, 2002), is missing in plants.

Developmental stability of nucleolar dominance was investigated in hybrids of *Brassica* and *Solanum*. Classical experiments in *Brassica napus* allotetraploids showed developmental lability of nucleolar dominance and partial reactivation of under-dominant genes in floral organs (Chen and Pikaard, 1997b). In the early 1990s RV in the VH group employed, perhaps for the first time, quantitative RT-PCR for the analysis of nucleolar dominance in plants. Using this method, they determined the levels of homoelogous ETS transcripts in different organs of tomato x potato hybrids showing a strong nucleolar dominance of tomato genes in leaf but not in anthers and calli (Komarova et al., 2004). Activation of partner units was apparently linked to changes in DNA methylation and chromatin organization. Indeed, profound changes in condensation of rDNA chromatin were observed between tobacco leaf and root (Koukalova et al., 2005).

Earlier cytogenetic data from *N. tabacum* indicated that unconverted parental units of *N. sylvestris*-origin were highly methylated, perhaps located at a locus on chromosome S12 (Lim et al., 2000) that does not show secondary constrictions at metaphase (a hallmark of genetic inactivity). More recently (Dadejova et al., 2007) used RT-PCR to investigate expression of rRNA genes in a number of synthetic *Nicotiana* hybrids (including reciprocal crosses) with a genomic composition similar to natural *N. tabacum* (SSTT), *N. rustica* (PPUU) and *N. arentsii* (UUWW) allotetraploids differing in age and genome donors. They found strong uniparental rDNA silencing of *N. paniculata* genes in *N. paniculata* × *N. undulata* F1 hybrids (genome composition corresponding to natural *N. rustica*), whereas *N. sylvestris* × *N. tomentosiformis* (*N. tabacum*) and *N. undulata* × *N. wigandioides* (*N. arentsii*) F1 hybrids showed little or no silencing (i.e., co-dominance). Based on these observations, Kovarik et al. (2008) proposed that nucleolar dominance, established early in allopolyploid formation including F1 hybrids, plays a significant role in further molecular evolution of rDNA. It has been suggested that epigenetic silencing of rDNA loci makes them less vulnerable to homogenization and more likely to be lost, perhaps thousands or millions of years later.

In 2003, AK visited the VH lab in Tübingen. At that time, both groups were fascinated by the dynamics of repetitive sequences, especially their species- and sometimes even population-specific features. As a result of fruitful discussions during a stroll around the old castle (whose walls remember the discovery of DNA by Friedrich Miescher) we wrote a review paper on the behavior of satellite DNA repeats in plant hybrids (Hemleben et al., 2007). The outcome was a productive collaborative research on allopolyploidy carried out in labs at the University of Tübingen, Queen Mary College of the University of London and the Czech Academy of Science. The findings are significant for our understanding of evolution of plant species since the world of angiosperms is largely dominated by allopolyploids.

## Concluding Remarks

Looking back over 50 years of research on rDNA, it is amazing to see how many complex factors stimulated or supported this field of study. To start with: In the early 1960s, the interest in molecular biology was rising: Basic functions of cell organelles were clarified. Electron microscopy, ultracentrifugation, radioactive labelling, and gel electrophoresis and hybridization assays were the key experimental methods, revealing a complex and highly organized ribosome construction and assembly process. As in bacteria, but with variation in size in all eukaryotes, the 40S subunit were shown to contain 18S rRNA while the 60S subunit associates with 25/28S (plants/animals), 5.8S and a smaller 5S rRNA. Both subunits form a ribonucleoprotein complex which assembles into a functional 80S ribosome. Transcription of the 18S, 5.8S and 25S was found to occur by RNA polymerase I as a large polycistronic precursor (pre-rRNA) containing tandem repeat sequences of the 18S-5.8S-25S rDNA multigene family, which is subsequently processed into the mature rRNAs, whereas the 5S rRNA genes (5S rDNA) are transcribed by RNA polymerase III. Gene technologies, gene cloning and DNA sequencing showed that strongly conserved parts alternate with more variable regions, especially in the intergenic regions of these multigene families. Cooperation of various experts delivered further valuable information and opened up the broad field of plant molecular phylogeny, molecular evolution and molecular systematics, supported by the rapidly growing field of whole genome sequencing coupled with more and more sophisticated computer evaluation of the data obtained. New techniques of cytogenetics made genome evolution and species formation visible. Especially for plants, the process of homo- and allopolyploidy by natural hybridization could be followed. The phenomenon of nucleolar dominance helped us to understand the mechanisms of silencing of rDNA from one partner probably leading to elimination of rRNA genes in allopolyploids.

## Author Contributions

VH conceived and designed the study. AK, DG, NB, RV, and VH contributed to the writing of the manuscript. All authors contributed to the article and approved the submitted version.

## Funding

AK: The work was supported by the Czech Science Foundation (grant 19-03442S). VH: Our work was always supported by the German Science Foundation (DFG), some parts by the Federal Ministry of Research and Technology (BMFT), today called BMBF; the Alexander v. Humboldt Foundation (Bonn, Germany) greatly supported the research stay of NB and RV at Tübingen University which is gratefully acknowledged. DG is grateful to former UK governments for financial support during BSc studies at University of East Anglia and PhD (Science & Industry Award) at the University of Edinburgh and to the University of Nottingham. RA is grateful to the Ministry of Education and Science of Ukraine, the Alexander von Humboldt Foundation, German Academic Exchange Service (DAAD) and Austrian Exchange Service (OeAD) for the financial support provided to him and members of his research group. NB is grateful for the earlier support from the Alexander von Humboldt Foundation; he is currently supported by a personal grant from the Huaiyin Normal University (Huai’an, China).

## Conflict of Interest

The authors declare that the research was conducted in the absence of any commercial or financial relationships that could be construed as a potential conflict of interest.

## Publisher’s Note

All claims expressed in this article are solely those of the authors and do not necessarily represent those of their affiliated organizations, or those of the publisher, the editors and the reviewers. Any product that may be evaluated in this article, or claim that may be made by its manufacturer, is not guaranteed or endorsed by the publisher.
